# Analysis of Social Media Use, Mental Health, and Gender Identity Among US Youths

**DOI:** 10.1001/jamanetworkopen.2023.24389

**Published:** 2023-07-24

**Authors:** Sarah M. Coyne, Emily Weinstein, J. Andan Sheppard, Spencer James, Megan Gale, Megan Van Alfen, Nora Ririe, Cameron Monson, Sarah Ashby, Allison Weston, Kennedy Banks

**Affiliations:** 1 School of Family Life, Brigham Young University, Provo, Utah; 2 Harvard Graduate School of Education, Cambridge, Massachusetts

## Abstract

**Importance:**

Mental health among children and adolescents is a critical public health issue, and transgender and gender nonbinary youths are at an even greater risk. Social media has been consistently associated with youth mental health, but little is known about how gender identity interacts with this association.

**Objective:**

To use a risk and resilience approach to examine the association between social media use and mental health among transgender, gender nonbinary, and cisgender youths.

**Design, Setting, and Participants:**

This cross-sectional study analyzed data collected from an online survey between May and August 2021. Participants included a random sample of US youths; eligibility requirements included being aged 10 to 17 years and residing in the US. Statistical analysis was performed from February to April 2022.

**Main Outcomes and Measures:**

Social media use (time, type of use, favorite site, social comparisons, mindfulness, taking intentional breaks, cleaning and curating feeds, problematic use, and media literacy programs at their school) and mental health (depression, emotional problems, conduct problems, and body image) as main outcomes.

**Results:**

Participants included 1231 youths aged 10 to 17 years from a national quota sample from the United States; 675 (54.8%) identified as cisgender female, 479 (38.9%) as cisgender male, and 77 (6.3%) as transgender, gender nonbinary, or other; 4 (0.3%) identified as American Indian or Alaska Native, 111 (9.0%) as Asian, 185 (15.0%) as Black, 186 (15.1%) as Hispanic or Latinx, 1 (0.1%) as Pacific Islander, 703 (57.1%) as White, and 41 (3.3%) as mixed and/or another race or ethnicity. Gender identity moderated both the strength and the direction of multiple associations between social media practices and mental health: active social media use (eg, emotional problems: *B* = 1.82; 95% CI, 0.16 to 3.49; *P* = .03), cleaning and/or curating social media feeds (eg, depression: *B* = −0.91; 95% CI, −1.98 to −0.09; *P* = .03), and taking intentional breaks (eg, depression: *B* = 1.03; 95% CI, 0.14 to 1.92; *P* = .02).

**Conclusions and Relevance:**

In this cross-sectional study of gender identity, social media, and mental health, gender identity was associated with youths’ experiences of social media in ways that may have distinct implications for mental health. These results suggest that research about social media effects on youths should attend to gender identity; directing children and adolescents to spend less time on social media may backfire for those transgender and gender nonbinary youths who are intentional about creating safe spaces on social media that may not exist in their offline world.

## Key Points

**Question:**

Is gender identity associated with social media use and mental health among youths?

**Findings:**

This cross-sectional study of 1231 transgender, gender nonbinary, and cisgender youths found that gender identity moderated both the effect size and the direction of the association between social media use and mental health.

**Meaning:**

These results suggest that gender identity is a key moderator when examining youth social media use and mental health and should be included in studies moving forward.

## Teaser

This cross-sectional study examines the association between social media use and mental health among transgender, gender nonbinary, and cisgender youths in the US.

## Introduction

The percentage of transgender and nonbinary (TGNB) youths coming out in the US has doubled in the last decade,^1^ with 1.4% as transgender and 3.0% as nonbinary.^2^ Despite studies documenting an association between gender, social media use (SMU), and indicators of mental health,^3,4,5^ and that TGNB youths are at a higher risk for mental health issues than cisgender youths,^6,7,8^ relatively little research has examined the association between SMU and mental health among TGNB youths.^9^ Consequently, this study aimed to directly explore the interplay between gender identity, SMU, and indicators of mental health, including internalizing (emotional problems, depression, and body image), and externalizing problems (conduct problems).

Gender identity is an individual’s deeply felt sense of being a man, woman, or an alternate gender (eg, nonbinary), which sometimes may not align with the sex assigned at birth.^10^ TGNB individuals may experience gender dysphoria, an intense distress because of the disconnect between one’s assigned sex and internal gender identity.^10,11^ TGNB individuals may also experience high levels of minority stress,^12,13^ the stress of being a minority in a majority social environment^14,15,16^ fostered through social processes, institutions, and structures that harass and/or discriminate.^13,17^ For TGNB youths, minority stress can manifest through violence due to gender nonconformity,^18,19,20^ gender dysphoria,^21^ family tension, and emotional distress from fear of rejection^21,22^ and is associated with increased mental health struggles and greater risk for suicide.^17,23,24,25^ Currently, 25% to 32% of TGNB youths attempt suicide.^18,26^ Though there are public concerns about the effects of SMU on adolescent mental health, research on TGNB youths suggests social media may be a protective factor instead of a risk for mental health.^9^

The research on SMU and mental health tends to be mixed. Some studies suggest time spent on social media is associated with mental health problems,^4,27,28^ while others find no link.^29,30,31^ Certain variables may moderate the association between SMU and mental health, including SMU context and content, problematic behaviors,^32^ and gender (associations greater for girls than boys^33^), suggesting gender identity as a potential moderator.^34^ School media literacy may also help youths use social media in ways that might benefit their mental health; however, this is understudied with gender minority youths.^35^

TGNB individuals engage with media in various ways for multiple reasons.^9,12^ While general media represents TGNB individuals less frequently and accurately than cisgender individuals,^36,37,38,39^ social media allows TGNB individuals to portray themselves how they see fit. TGNB youths access social media for a variety of content, building positive connections^38^ and creating support systems protective against mental illness, based on common interests and experiences.^9,40,41,42^ Little is known, however, about how TGNB youths use social media in general outside of gender identity exploration and development.

Acknowledging continued interest in the association between social media and mental health, elevated mental health risks relevant to TGNB youths, and the potential effects of social media in the lives of TGNB youths, our study aimed to examine the association between SMU and mental health as moderated by gender identity. Following minority stress theory, we hypothesized that positive social media practices may be more protective for TGNB youths (compared with cisgender youths) as it may reduce feelings of minority stress.

## Methods

The survey was approved by the Brigham Young University institutional review board and participants were treated under the human participants’ guidelines from the American Psychological Association. Informed consent was obtained online. Parents gave consent for their minor children to participate. This study followed the Strengthening the Reporting of Observational Studies in Epidemiology (STROBE) reporting guideline for cross-sectional studies by having multiple TGNB individuals as authors and addressing potential bias via qualitative focus groups with TGNB youths.

Participants included a national quota sample of 1231 youths aged 10 to 17 years obtained using a Qualtrics panel and collected between May and August 2021. The panel consisted of youths from all 50 states. We gave Qualtrics quotas for race and ethnicity based on recent US Census data, and they recruited based on those estimates. We chose this age group to examine because many youths begin using social media during this age. Additionally, there is substantial gender identity development during adolescence.

Race and ethnicity were self-reported; categories included American Indian or Alaska Native, Asian, Black, Hispanic or Latinx, Pacific Islander, White, and mixed or another race or ethnicity. Self-reported gender categories included cisgender female, cisgender male, and transgender, nonbinary, or other. Household income data were also collected.

### Measures

The data were skewed for outcome measures. Descriptions of transformations and reliability statistics are included in Table 1.

**Table 1.** Description and Reliability of Measures for Adolescent Outcomes and Social Media Practices by Gender Identity

Variables	Reliability, Cronbach α	Mean (SD)			*F* value	*P* value	Effect size (partial η^2^)
		Female	Male	TGNB			
Outcome							
Depression^a^	0.93	2.22 (0.87)	2.07 (0.93)	2.80 (0.91)	22.54	<.001	0.035
Emotional problems^b^	0.84	1.98 (0.59)	1.72 (0.63)	2.33 (0.54)	45.96	<.001	0.070
Conduct problems^c^	0.71	1.52 (0.45)	1.60 (0.50)	1.68 (0.46)	6.64	<.001	0.011
Body image^d^	0.88	3.00 (1.01)	3.52 (0.83)	2.26 (0.91)	79.87	<.001	0.115
Social media variables							
Social media time	NA	3.22 (2.00)	3.05 (2.05)	2.82 (1.73)	1.61	.20	0.003
Age at first smartphone	NA	11.25 (1.99)	11.28 (2.26)	11.25 (2.09)	0.31	.74	0.001
Active use	0.65	2.97 (0.95)	3.45 (1.00)	2.98 (0.90)	30.74	<.001	0.057
Passive use	NA	3.74 (1.16)	3.77 (1.11)	3.74 (1.18)	0.09	.91	0.000
Problematic social media use	0.83	1.13 (0.45)	1.51 (0.56)	1.12 (0.57)	67.35	<.001	0.117
School media literacy	NA	2.92 (1.31)	3.60 (1.25)	2.15 (0.57)	51.15	<.001	0.090
Intentional breaks	NA	4.93 (1.20)	4.99 (1.18)	4.92 (1.19)	0.26	.77	0.001
Social comparisons	0.82	2.55 (1.06)	2.94 (1.24)	2.67 (1.08)	13.37	<.001	0.026
Cleaning and/or curating feed	0.84	3.29 (1.40)	3.41 (1.50)	3.70 (1.39)	3.757	.02	0.007
Mindful social media use	0.82	3.34 (1.34)	3.25 (1.28)	3.38 (1.45)	0.644	.53	0.001

Abbreviations: NA, not applicable; TGNB, transgender and nonbinary.

^a^ For transformations of depression outcome variables, the low depression group was 0 to 1.9, and the high depression group was 2.0 to 4.0.

^b^ For transformations of emotional problem outcome variables, the low emotional problem group was 0 to 1.9, and the high emotional problem group was 2.0 to 3.0.

^c^ For transformations of conduct problem outcome variables, the low conduct problem group was 0 to 1.9, and the high conduct problem group was 2.0 to 3.0.

^d^ For transformations of body image outcome variables, the low and/or moderate body image group was 1.0 to 2.9, and the high body image group was 3.0 to 5.0.

#### Outcome Variables

##### Depression

The patient health questionnaire (PHQ-8) measured participant depression.^43^ Youths reported how frequently they experienced 8 symptoms within the last 2 weeks. A sample item of the PHQ-8 included “Feeling down, depressed, or hopeless.” Responses ranged from 1 (not at all) to 4 (nearly every day).

##### Emotional Problems

Five items measuring emotional problems were completed from the Strengths and Difficulties Questionnaire (SDQ).^44^ Items were rated on a 3-point scale, 1 (not true) to 3 (certainly true), and a sample item included “I worry a lot.”

##### Conduct Problems

Five items measuring conduct problems from the SDQ were also completed. The same rating scale was used, and a sample item included, “I take things that are not mine.”

##### Body Image

Youths reported how often they agreed with 3 statements about body image^45^ using a 5-point Likert-scale: 1 (never) to 5 (always). A sample item included, “I’m pretty happy about the way I look.”

#### Media Variables

##### Social Media Time

Participants were first asked if they had ever used social media. Seventy-two participants (5.8%) reported they had never used social media and were omitted from all future analyses. Participants who reported they used social media estimated the hours they spent on social media in a typical day. This was measured on a scale of 1 (none) to 8 (more than 8 hours). Though only moderate indicators of SMU, self-reports of screen time provide information about youths’ perceptions of their screen time and differentiates between light and heavy users.^46^

##### Age at First Smartphone

For youths who had a smartphone, they were asked how old they were when they got their first smartphone. These answers ranged from age 5 to 17 years.

##### Active or Passive Use

Youths were asked how often they participated in certain habits while on social media to determine if they were active or passive social media users. Three items measured active use (eg, “Make comments or like other people’s posts”), while 1 item measured passive use (“Mostly scroll through other people’s posts without commenting or posting myself”). Responses were measured on a 5-point Likert scale, 1 (never) to 5 (all the time).

##### Social Media Comparison

Youths were asked to report the frequency of 3 social comparison behaviors when visiting their most used social media site^47^ on a 5-point Likert-type scale, 1 (never) to 5 (always). A sample item included, “Compare my life with other people’s lives.”

##### Taking Intentional Breaks

Youths were asked how often they take intentional breaks from their smartphones (eg, by putting their smartphone on airplane mode or leaving it in another room). Responses were measured on a 6-point Likert scale, 1 (never or rarely) to 6 (every day or almost every day).

##### Problematic SMU

Youths were first asked if they used either social media or video games more frequently. Then, youths who reported that they used social media more responded how much they agreed with 7 items related to their social media habits (those who chose video games were excluded from this scale only). This scale was adapted^48^ from a scale that originally assessed problematic cell phone use.^49^ Participants were asked to rate how much they agreed with a series of statements regarding their SMU (eg, “When I am not using social media, I am thinking about using it or planning the next time I can use it”). Items were rated using a 5-point Likert scale, 1 (strongly disagree) to 5 (strongly agree).

##### Digital Well-Being in Schools

Youths rated on a 5-point Likert scale how much they agreed with the statement, “My school tries to help us learn how to use our phones or social media in healthy ways.” Items were rated from 1 (strongly disagree) to 5 (strongly agree).

##### Mindful Media Use

A modified Mindfulness Attention Awareness Scale^50^ was used to measure mindfulness around SMU. Youths were asked to think about the last time they were on social media and how much certain behaviors were present (eg, “I was engaging with social media without really paying attention”). Items were rated on a 6-point Likert scale, 1 (not at all) to 6 (very much). This was recoded so a higher score indicated more mindful media use.

##### Cleaning and Curating

Participants were asked 2 items about how regularly they cleaned or curated their social media feed or followers (eg, by muting or unfollowing certain accounts). Items were rated on a 6-point Likert scale, 1 (never) to 6 (about once a week).

#### Qualitative

Member checking is often considered a hallmark of careful and culturally considerate research.^35^ To aid with interpretation of the quantitative results, 7 adolescents were recruited as an advisory board via 2 focus groups centered on cointerpreting the findings. The advisory group was not designed for further data collection nor to change study results. Rather, the process emphasized cointerpretation with adolescents who have relevant lived experience, allowing insider perspectives to expand the interpretation and potential directions for future research.

The first group consisted of 4 cisgender adolescents aged 14 to 17 years and the second of 3 TGNB adolescents aged 14 to 16 years, all residing in the US. Both focus groups took place over a video call and were led by 1 cisgender and 2 TGNB adults while a team of 4 individuals assisted in note taking. After brief introductions, group facilitators presented the survey findings and asked the participants to provide their interpretations and asked follow-up questions for clarity.

### Statistical Analysis

Basic descriptive statistics were first conducted for all major variables based on gender identity. This was done using multivariate analysis of variance (MANOVA). We then conducted 4 logistic regressions, with outcomes being emotional problems, depression, conduct problems, and body image. Independent variables assessed included social media time and the aforementioned contextual social media factors. Covariates included race, age, income, and family structure. We also explored gender identity as a moderator in each model. Sex as a biological variable correlated highly with gender identity and was not included as a covariate. Statistical significance was determined at 2-sided *P* < .05. Missing data were handled using the maximum likelihood method in Mplus (Muthén & Muthén). Statistical analysis was performed from February to April 2022 using Stata version 17 (StataCorp).

## Results

Participants included 1231 youths from a national quota sample from the United States; 675 (54.8%) identified as cisgender female, 479 (38.9%) as cisgender male, and 77 (6.3%) as transgender, gender nonbinary, or other; 4 (0.3%) identified as American Indian or Alaska Native, 111 (9.0%) as Asian, 185 (15.0%) as Black, 186 (15.1%) as Hispanic or Latinx, 1 (0.1%) as Pacific Islander, 703 (57.1%) as White, and 41 (3.3%) as mixed and/or another race or ethnicity; age ranged from 10 to 17 years with a mean (SD) of 14.5 (2.0) years. Average household income was between $60 000 and $75 000 per year (with 308 [25.0%] below $50 000 per year and 431 [35.0%] above $100 000 per year).

### Preliminary Analyses

A series of MANOVAs explored gender identity differences in outcomes and SMU. There was a significant multivariate effect of gender identity differences for all outcomes measured in the study (*F*_8, 2452_ = 30.32; *P* < .001; η^2^ = 0.09). TGNB youths had the highest levels of depression, emotional problems, conduct problems, and the worst body image compared with other youths (eg, mean [SD] depression measures were 2.22 [0.87] for female, 2.07 [0.93] for male, and 2.80 [0.91] for TGNB; *P* < .001). See Table 1 for full statistics and mean comparisons.

For media variables, cisgender male youths tended to have higher levels of active SMU, problematic SMU, and social comparisons than cisgender female youths or TGNB youths (eg, mean [SD] active SMU measures were 2.97 [0.95] for female, 3.45 [1.00] for male, and 2.98 [0.90] for TGNB; *P* < .001); they also perceived having schools with stronger digital literacy programs. Additionally, TGNB youths reported higher levels of cleaning or curating their social media feed than other youths (mean [SD] level of cleaning and/or curating social media feeds were 3.29 [1.40] for female, 3.41 [1.50] for male, and 3.70 [1.39] for TGNB; *P* < .001) (Table 1).

### Main Analyses

In general, time spent on social media and age at receiving first smartphone were not associated with any outcomes. Attending a school with what students perceived as strong digital literacy training, active SMU, low levels of social comparisons, and low levels of problematic SMU (eg, depression: *B* = 1.02; 95% CI, 0.66-1.38; *P* < .001) were associated with lower risk of mental health problems (Table 2). Four results were significantly moderated by gender identity; given the focus of the study, we focus on these 4 results.

**Table 2.** Logistic Regression for Social Media Variables and Mental Health Outcomes^a^

Social media variables	Outcomes											
	Depression			Emotional problems			Conduct problems			Body image		
	*B *(95% CI)	SE	*P* value	*B *(95% CI)	SE	*P* value	*B *(95% CI)	SE	*P* value	*B *(95% CI)	SE	*P* value
Age at first smart phone	−0.09 (−0.42 to 0.25)	0.17	.61	0.01 (− 0.31 to −0.33)	0.16	.97	−0.25 (−0.63 to −0.12)	0.19	.19	0.17 (−0.16 to −0.51)	0.17	.30
Gender identity	1.06 (−.90 to 3.02)	1.00	.29	−1.36 (−3.25 to −0.52)	0.96	.16	−1.61 (−3.87 to −0.64)	1.15	.16	−0.72 (−2.88 to −1.33)	0.26	.47
Social media time	0.02 (−0.07 to 0.11)	0.05	.69	−.03 (−0.13 to −0.05)	0.05	.40	−0.01 (−0.12 to −0.09)	0.05	.80	0.01 (−0.08 to −0.09)	0.04	.84
Active use	−0.04 (−0.25 to 0.19)	0.11	.78	−0.18 (−0.40 to −0.04)	0.11	.10	0.05 (−0.20 to −0.31)	0.13	.68	0.46 (0.24 to −0.68)	0.11	<.001
Passive use	0.10 (−0.06 to 0.27)	0.08	.21	0.07 (−0.09 to −0.22)	0.08	.38	0.04 (−0.15 to −0.24)	0.10	.65	−0.08 (−0.24 to −0.08)	0.08	.32
Social comparisons	0.52 (0.31 to 0.73)	0.11	.001	0.48 (0.29 to −0.68)	0.10	<.001	0.54 (0.31 to −0.76)	0.11	<.001	−0.48 (−0.67 to −0.28)	0.10	<.001
Intentional breaks	−0.11 (−0.11 to 0.08)	0.08	.16	−0.15 (−0.30 to −0.01)	0.08	.049	−0.12 (−0.29 to −0.05)	0.09	.16	0.13 (−0.02 to −0.27)	0.13	.09
School digital literacy	−0.19 (−0.19 to 0.08)	0.08	.02	−0.10 (−0.24 to −0.04)	0.07	.17	0.01 (−0.16 to −0.18)	0.09	.91	0.29 (0.15 to −0.44)	0.07	<.001
Clean/curate feed	0.48 (0.33 to 0.64)	0.08	<.001	0.31 (0.17 to −0.45)	0.07	<.001	0.29 (0.12 to −0.44)	0.08	<.001	−0.08 (−0.22 to −0.05)	0.07	.23
Mindful media	−0.04 (−0.15 to 0.07)	0.06	.47	−0.05 (−0.17 to 0.05)	0.06	.31	−0.04 (−0.17 to −0.08)	0.06	.51	0.01 (−0.10 to −0.12)	0.06	.90
Problematic social media use	1.02 (0.66 to 1.38)	0.18	<.001	0.41 (0.10 to −0.72)	0.16	.009	0.49 (0.10 to −0.87)	0.19	.01	−0.06 (−0.36 to −0.24)	0.15	.69

^a^ Only logistic regression main effect sizes are shown in the table for parsimony. *B* refers to the unstandardized coefficient (point estimate).

**Figure 1.**
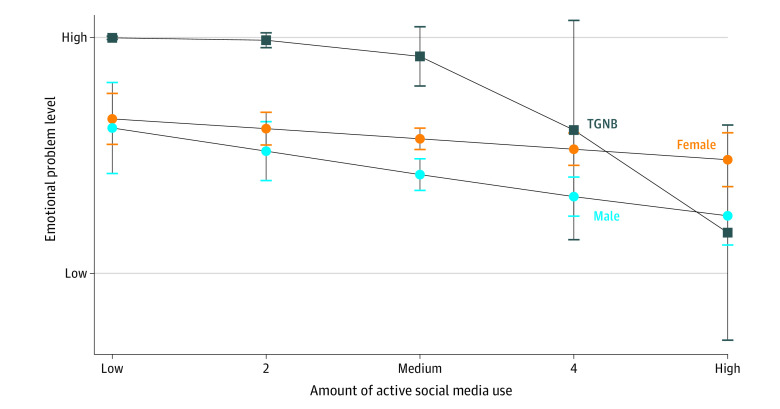
Active Social Media Use and Emotional Problems by Gender Identity TGNB indicates transgender and nonbinary youths.

**Figure 2.**
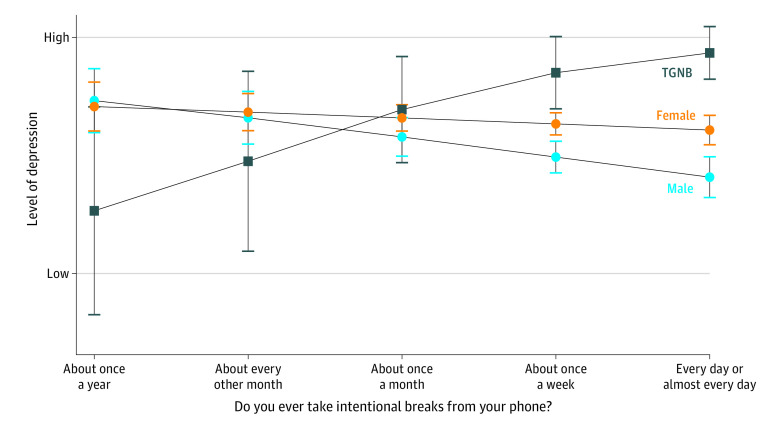
Intentional Phone Breaks and Depression by Gender Identity TGNB indicates transgender and nonbinary youths.

**Figure 3.**
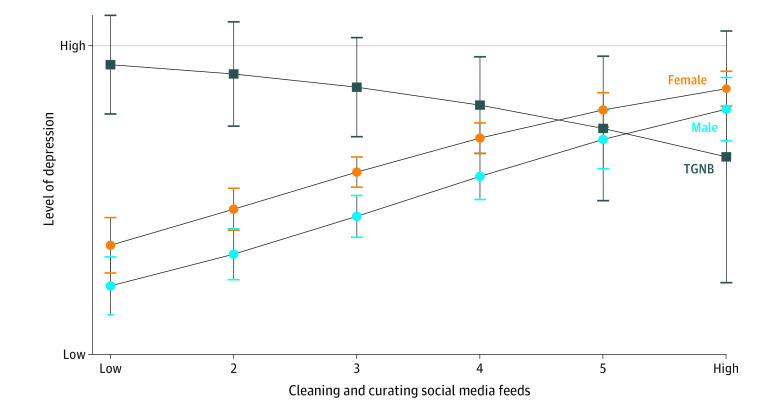
Cleaning and Curating Social Media Feeds and Depression by Gender Identity TGNB indicates transgender and nonbinary youths.

First, there was a significant interaction between gender identity and active SMU for emotional problems (*B* = 1.82; 95% CI, 0.16 to 3.49; *P* = .03). Specifically, active media use was more associated with lower emotional problems for TGNB youths than for cisgender youths (Figure 1). Second, there was a significant moderation between gender identity and taking intentional breaks for depression (*B* = 1.03; 95% CI, 0.14 to 1.92; *P* = .02) and emotional problems (*B* = 1.51; 95% CI, 0.37 to 2.65; *P* = .009). Taking intentional social media breaks was positively associated with depression for TGNB, but negatively associated with cisgender participants (Figure 2). Third, there was a significant moderation between gender identity and cleaning or curating feeds for depression (*B* = −0.91; 95% CI, −1.98 to −0.09; *P* = .03) and conduct problems (*B* = −0.64; 95% CI, −1.18 to −0.11; *P* = .02). Specifically, depression and conduct problems were lower for TGNB youths when they reported regularly cleaning or curating their social media feed, but both depression and conduct disorders were higher for cisgender youths when they engaged in this same activity (Figure 3). Finally, we found a significant interaction between school media literacy and gender identity for depression (*B* = −1.07; 95% CI, −1.98 to −0.15; *P* = .02) with school media literacy being more associated with lower rates of depression for TGNB youths vs cisgender youths.

## Discussion

There were significant gender identity differences for all health outcomes measured in the study. TGNB youths had the highest levels of depression, emotional problems, conduct problems, and negative body image compared with cisgender youths. However, TGNB youths’ use of social media was differentially associated with mental health.

Aligning with previous research on SMU,^51,52^ this study found that active media use was associated with lower rates of mental health problems, especially for TGNB youths. As our advisory board suggested, TGNB youths may be more intentional about creating online spaces that are free from the negative interactions that can plague them in school^53^ or at home.^9^ One TGNB youth shared, “On social media, I am able to choose to be around the people that don’t make me uncomfortable, that don’t make me hate myself.” TGNB youths can actively be themselves (present themselves) in a way that aligns with their identity via pictures and pronouns.

Relatedly, cleaning and/or curating feeds was associated with lower levels of depression for TGNB youths but higher levels for cisgender youths. TGNB youths are more likely to be bullied or harassed online and offline and may therefore have a greater need to curate safe spaces for themselves on social media. Illustrating this point, one TGNB youth said, “Real life isn’t safe for LGBTQ people, but online there is more control where I can find people who have similar beliefs.” Conversely, cleaning and/or curating feeds appeared to have an opposite pattern for cisgender youths, a finding which merits further investigation: perhaps cisgender youths fear offending peers by unfollowing those in their social circle, and thus benefit from a social media break by uninstalling apps entirely rather than curating feeds.

Taking intentional technology breaks was significantly associated with increases in depression and emotional problems for TGNB youths but not for cisgender youths, again suggesting the balance of risks and benefits of youths’ SMU differs by gender identity. For TGNB youths for whom social media is a key venue for social acceptance, breaks could cut this off and potentially be detrimental to health.^9^ As a TGNB advisory board adolescent explained, “I’m fine taking breaks because I already have a support group that is super nice to me. For others, when they delete it, [they delete] their safe place. That’s why they feel bad…they don’t have that community anymore.”

Attending a school with a perceived strong media literacy program was also associated with positive outcomes for all youths,^54,55^ and again particularly so for TGNB youths. Given the apparent importance of online spaces for TGNB youths, these programs may contribute to protective practices and facilitate even greater intentionality around SMU.^38^

### Implications

Parents could be less concerned about screen time potentially causing mental health struggles in their TGNB youth and instead focus on how social media may be a resource for their children in the face of everyday minority stress. Policy makers and school officials worried about the link between social media and mental health should consider the differential associations of social media by gender identity and take a more person-centered approach. Blanket policies that severely limit SMU among youths may have different (and more negative) impacts for TGNB youths. We encourage policies (at school, state, and national levels) that focus on supporting school media literacy programs as opposed to only limiting screen time. Pediatricians might consider asking detailed questions around media use beyond screen time at well-child checkups. Clinical and medical professionals treating adolescents might consider discussing social media practices and may take a more nuanced approach depending on patient gender identity.

### Limitations

This study had limitations. Major limitations included the self-report and cross-sectional nature of the data, and there were a relatively low number of TGNB youths.

## Conclusions

The association between social media and mental health is complex and nuanced. The present findings indicate that TGNB youths are at an elevated risk for negative health outcomes compared with cisgender youths.^56,57^ These differences do not seem to reflect their time on social media. Rather, SMU appears to be associated with lower levels of mental health problems for TGNB youths,^9^ reaffirming that person-specific differences are key when examining social media and health and pointing to the importance of deliberate attention to gender identity.^58^ Although TGNB youths are among the highest risk for mental health struggles and suicidality, social media might be protective for some TGNB youths, particularly when used in protective ways.

## Notes

Supplement.
Data Sharing Statement

## References

[1] Herman JL, Flores AR, O’Neill KK. How many adults and youth identify as transgender in the United States? June 2022. Accessed January 25, 2023. https://williamsinstitute.law.ucla.edu/wp-content/uploads/Trans-Pop-Update-Jun-2022.pdf

[2] Brown A. About 5% of young adults in the U.S. say their gender is different from their sex assigned at birth. Pew Research Center. June 7, 2022. Accessed January 25, 2023. https://pewrsr.ch/3Qi2Ejd

[3] Orben A, Przybylski AK, Blakemore SJ, Kievit RA. Windows of developmental sensitivity to social media. Nat Commun. 2022;13(1):1649. doi:10.1038/s41467-022-29296-3 35347142 PMC8960761

[4] Twenge JM, Joiner TE, Rogers ML, . Increases in depressive symptoms, suicide-related outcomes, and suicide rates among US adolescents after 2010 and links to increased new media screen time. Clin Psychol Sci. 2017;6(1):3-17. doi:10.1177/2167702617723376

[5] Valkenburg PM, Peter J. The differential susceptibility to media effects model. J Commun. 2013;63(2):221-243. doi:10.1111/jcom.12024

[6] Aparicio-García ME, Díaz-Ramiro EM, Rubio-Valdehita S, López-Núñez MI, García-Nieto I. Health and well-being of cisgender, transgender and non-binary young people. Int J Environ Res Public Health. 2018;15(10):2133. doi:10.3390/ijerph15102133 30274141 PMC6209926

[7] Grossman AH, D’augelli AR. Transgender youth: invisible and vulnerable. Current Issues in Lesbian, Gay, Bisexual, and Transgender Health. J Homosex. Published online October 17, 2008. doi:10.1300/J082v51n01_06

[8] Grossman AH, Frank JA, McCutcheon MJ. Sexual orientation and aging in Western society. In: Patterson CJ, D’Augelli AR, eds. Handbook of Psychology and Sexual Orientation. Oxford University Press; 2023:132-148. doi:10.1093/acprof:oso/9780199765218.003.0010.

[9] Fish JN, McInroy LB, Paceley MS, . “I’m kinda stuck at home with unsupportive parents right now”: LGBTQ youths’ experiences with COVID-19 and the importance of online support. J Adolesc Health. 2020;67(3):450-452. doi:10.1016/j.jadohealth.2020.06.002 32591304 PMC7309741

[10] American Psychiatric Association. What is gender dysphoria? 2016. Accessed October 19, 2020. https://www.psychiatry.org/patients-families/gender-dysphoria/what-is-gender-dysphoria

[11] Hunt QA, Morrow QJ, McGuire JK. Experiences of suicide in transgender youth: a qualitative, community-based study. Arch Suicide Res. 2020;24(sup2):S340-S355. doi:10.1080/13811118.2019.1610677 31062669

[12] Lefevor GT, Boyd-Rogers CC, Sprague BM, Janis RA. Health disparities between genderqueer, transgender, and cisgender individuals: an extension of minority stress theory. J Couns Psychol. 2019;66(4):385-395. doi:10.1037/cou0000339 30896208

[13] Meyer IH. Prejudice, social stress, and mental health in lesbian, gay, and bisexual populations: conceptual issues and research evidence. Psychol Bull. 2003;129(5):674-697. doi:10.1037/0033-2909.129.5.674 12956539 PMC2072932

[14] Meyer IH. Minority stress and mental health in gay men. J Health Soc Behav. 1995;36(1):38-56. doi:10.2307/2137286 7738327

[15] Ross CE, Mirowsky J. Explaining the social patterns of depression: control and problem solving–or support and talking? J Health Soc Behav. 1989;30(2):206-219. doi:10.2307/2137014 2738367

[16] Pearlin LI. The sociological study of stress. J Health Soc Behav. 1989;30(3):241-256. doi:10.2307/2136956 2674272

[17] Marshal MP, Friedman MS, Stall R, . Sexual orientation and adolescent substance use: a meta-analysis and methodological review. Addiction. 2008;103(4):546-556. doi:10.1111/j.1360-0443.2008.02149.x 18339100 PMC2680081

[18] Clements-Nolle K, Marx R, Katz M. Attempted suicide among transgender persons: the influence of gender-based discrimination and victimization. J Homosex. 2006;51(3):53-69. doi:10.1300/J082v51n03_04 17135115

[19] Rood BA, Puckett JA, Pantalone DW, Bradford JB. Predictors of suicidal ideation in a statewide sample of transgender individuals. LGBT Health. 2015;2(3):270-275. doi:10.1089/lgbt.2013.0048 26788676 PMC4713016

[20] Testa RJ, Sciacca LM, Wang F, . Effects of violence on transgender people. Prof Psychol Res Pr. 2012;43(5):452-459. doi:10.1037/a0029604

[21] Bailey L, Ellis SJ, McNeil J. Suicide risk in the UK trans population and the role of gender transition in decreasing suicidal ideation and suicide attempt. Ment Health Rev (Brighton). 2014;19(4):209-220. doi:10.1108/MHRJ-05-2014-0015

[22] Kelleher C. Minority stress and health: implications for lesbian, gay, bisexual, transgender, and questioning (LGBTQ) young people. Couns Psychol Q. 2009;22(4):373-379. doi:10.1080/09515070903334995

[23] Mays VM, Cochran SD. Mental health correlates of perceived discrimination among lesbian, gay, and bisexual adults in the United States. Am J Public Health. 2001;91(11):1869-1876. doi:10.2105/AJPH.91.11.1869 11684618 PMC1446893

[24] Russell ST, Fish JN. Mental health in lesbian, gay, bisexual, and transgender (LGBT) youth. Annu Rev Clin Psychol. 2016;12:465-487. doi:10.1146/annurev-clinpsy-021815-093153 26772206 PMC4887282

[25] Ryan C, Russell ST, Huebner D, Diaz R, Sanchez J. Family acceptance in adolescence and the health of LGBT young adults. J Child Adolesc Psychiatr Nurs. 2010;23(4):205-213. doi:10.1111/j.1744-6171.2010.00246.x 21073595

[26] Grossman AH, D’Augelli AR. Transgender youth and life-threatening behaviors. Suicide Life Threat Behav. 2007;37(5):527-537. doi:10.1521/suli.2007.37.5.527 17967119

[27] Mérelle SYM, Kleiboer AM, Schotanus M, . Which health-related problems are associated with problematic video-gaming or social media use in adolescents? a large-scale cross-sectional study. Clinical Neuropsychiatry. Published February 2017. Accessed June 9, 2023. https://psycnet.apa.org/record/2017-10293-003

[28] Primack BA, Perryman KL, Crofford RA, Escobar-Viera CG. Social media as it interfaces with psychosocial development and mental illness in transitional-age youth. Child Adolesc Psychiatr Clin N Am. 2022;31(1):11-30. doi:10.1016/j.chc.2021.07.007 34801149

[29] Coyne SM, Rogers AA, Zurcher JD, Stockdale L, Booth M. Does time spent using social media impact mental health?: an eight year longitudinal study. Comput Human Behav. 2020;104:106160. doi:10.1016/j.chb.2019.106160

[30] Houghton S, Lawrence D, Hunter SC, . Reciprocal relationships between trajectories of depressive symptoms and screen media use during adolescence. J Youth Adolesc. 2018;47(11):2453-2467. doi:10.1007/s10964-018-0901-y 30046970 PMC6208639

[31] Orben A, Dienlin T, Przybylski AK. Social media’s enduring effect on adolescent life satisfaction. Proc Natl Acad Sci U S A. 2019;116(21):10226-10228. doi:10.1073/pnas.1902058116 31061122 PMC6534991

[32] Boer M, Stevens GWJM, Finkenauer C, de Looze ME, van den Eijnden RJJM. Social media use intensity, social media use problems, and mental health among adolescents: investigating directionality and mediating processes. Comput Human Behav. 2021;116:106645. doi:10.1016/j.chb.2020.106645

[33] Coyne SM, McDaniel BT, Stockdale LA. “Do you dare to compare?” associations between maternal social comparisons on social networking sites and parenting, mental health, and romantic relationship outcomes. Comput Human Behav. 2017;70:335-340. doi:10.1016/j.chb.2016.12.081

[34] Underwood MK, Ehrenreich SE. The power and the pain of adolescents’ digital communication: Cyber victimization and the perils of lurking. Am Psychol. 2017;72(2):144-158. doi:10.1037/a0040429 28221066 PMC5325156

[35] Weinstein E, James C. Behind Their Screens: What Teens Are Facing (and Adults Are Missing). MIT Press; 2022.

[36] Bouman WP, Arcelus J. The Transgender Handbook: A Guide for Transgender People, Their Families and Professionals. Nova Science Publishers; 2017.

[37] McLaren J, Bryant S, Brown B. “See me! recognize me!” an analysis of transgender media representation. Commun Q. 2021;69(2):172-191. doi:10.1080/01463373.2021.1901759

[38] McInroy LB, Craig SL. Transgender representation in offline and online media: LGBTQ youth perspectives. J Hum Behav Soc Environ. 2015;25(6):606-617. doi:10.1080/10911359.2014.995392

[39] Hughto JMW, Pletta D, Gordon L, Cahill S, Mimiaga MJ, Reisner SL. Negative transgender-related media messages are associated with adverse mental health outcomes in a multistate study of transgender adults. LGBT Health. 2021;8(1):32-41. doi:10.1089/lgbt.2020.0279 33170060 PMC7826438

[40] Selkie E, Adkins V, Masters E, Bajpai A, Shumer D. Transgender adolescents’ uses of social media for social support. J Adolesc Health. 2020;66(3):275-280. doi:10.1016/j.jadohealth.2019.08.011 31690534

[41] Evelyn S, Clancy EM, Klettke B, Tatnell R. A phenomenological investigation into cyberbullying as experienced by people identifying as transgender or gender diverse. Int J Environ Res Public Health. 2022;19(11):6560-6573. doi:10.3390/ijerph19116560 35682144 PMC9180504

[42] McInroy LB, Craig SL, Leung VWY. Platforms and patterns for practice: LGBTQ+ youths’ use of information and communication technologies. Child Adolesc Social Work J. 2019;36(5):507-520. doi:10.1007/s10560-018-0577-x

[43] Kroenke K, Spitzer RL, Williams JBW, Löwe B. An ultra-brief screening scale for anxiety and depression: the PHQ-4. Psychosomatics. 2009;50(6):613-621. doi:10.1176/appi.psy.50.6.613 19996233

[44] Goodman R, Ford T, Simmons H, Gatward R, Meltzer H. Using the Strengths and Difficulties Questionnaire (SDQ) to screen for child psychiatric disorders in a community sample. Br J Psychiatry. 2000;177:534-539. doi:10.1192/bjp.177.6.534 11102329

[45] Mendelson BK, Mendelson MJ, White DR. Body-esteem scale for adolescents and adults. J Pers Assess. 2001;76(1):90-106. doi:10.1207/S15327752JPA7601_6 11206302

[46] Hodes LN, Thomas KGF. Smartphone screen time: inaccuracy of self-reports and influence of psychological and contextual factors. Comput Human Behav. 2021;115:106616. doi:10.1016/j.chb.2020.106616

[47] Nesi J, Prinstein MJ. Using social media for social comparison and feedback-seeking: gender and popularity moderate associations with depressive symptoms. J Abnorm Child Psychol. 2015;43(8):1427-1438. doi:10.1007/s10802-015-0020-0 25899879 PMC5985443

[48] Stockdale LA, Coyne SM. Bored and online: reasons for using social media, problematic social networking site use, and behavioral outcomes across the transition from adolescence to emerging adulthood. J Adolesc. 2020;79:173-183. doi:10.1016/j.adolescence.2020.01.010 31978836

[49] Merlo LJ, Stone AM, Bibbey A. Measuring problematic mobile phone use: development and preliminary psychometric properties of the PUMP Scale. J Addict. 2013;2013:912807. doi:10.1155/2013/912807 24826371 PMC4008508

[50] Brown KW, West AM, Loverich TM, Biegel GM. Assessing adolescent mindfulness: validation of an adapted Mindful Attention Awareness Scale in adolescent normative and psychiatric populations. Psychol Assess. 2011;23(4):1023-1033. doi:10.1037/a0021338 21319908

[51] Rideout V. Measuring time spent with media: the Common Sense census of media use by US 8- to 18-year-olds. J Child Media. 2016;10(1):138-144. doi:10.1080/17482798.2016.1129808

[52] Thorisdottir IE, Sigurvinsdottir R, Asgeirsdottir BB, Allegrante JP, Sigfusdottir ID. Active and passive social media use and symptoms of anxiety and depressed mood among Icelandic adolescents. Cyberpsychol Behav Soc Netw. 2019;22(8):535-542. doi:10.1089/cyber.2019.0079 31361508

[53] Horne SG, McGinley M, Yel N, Maroney MR. The stench of bathroom bills and anti-transgender legislation: anxiety and depression among transgender, nonbinary, and cisgender LGBQ people during a state referendum. J Couns Psychol. 2022;69(1):1-13. doi:10.1037/cou0000558 34197153

[54] Cortesi S, Hasse A, Lombana-Bermudez A, Kim S, Gasser U. Youth and digital citizenship+ (plus): understanding skills for a digital world. SSRN. Preprint posted online April 14, 2020. doi:10.2139/ssrn.3557518

[55] Keles B, McCrae N, Grealish A. A systematic review: the influence of social media on depression, anxiety and psychological distress in adolescents. Int J Adolesc Youth. 2020;25(1):79-93. doi:10.1080/02673843.2019.1590851

[56] Delozier AM, Kamody RC, Rodgers S, Chen D. Health disparities in transgender and gender expansive adolescents: a topical review from a minority stress framework. J Pediatr Psychol. 2020;45(8):842-847. doi:10.1093/jpepsy/jsaa040 32626901

[57] Price-Feeney M, Green AE, Dorison S. Understanding the mental health of transgender and nonbinary youth. J Adolesc Health. 2020;66(6):684-690. doi:10.1016/j.jadohealth.2019.11.314 31992489

[58] Valkenburg P, Beyens I, Pouwels JL, van Driel II, Keijsers L. Social media use and adolescents’ self-esteem: heading for a person-specific media effects paradigm. J Commun. 2021;71(1):56-78. doi:10.1093/joc/jqaa039

