# Supplementary material for: Analysis of Social Media Use, Mental Health, and Gender Identity Among US Youths
Source: JAMA Netw Open. 2023 Jul 24;6(7):e2324389. doi: 10.1001/jamanetworkopen.2023.24389 (PMC10366700; doi:10.1001/jamanetworkopen.2023.24389)
Supplement: Supplement. — Data Sharing Statement [file jamanetwopen-e2324389-s001.pdf]

Data Sharing Statement

Coyne. Analysis of Social Media Use, Mental Health, and Gender Identity Among US Youths. *JAMA Netw Open*. Published July 24, 2023. doi:10.1001/jamanetworkopen.2023.24389

Data
Data available: Yes
Data types: Deidentified participant data
How to access data: Data is available on request by emailing smcoyne@byu.edu
When available: With publication

Supporting Documents
Document types: None

Additional Information
Who can access the data: Anyone requesting
Types of analyses: For scholarly purpose
Mechanisms of data availability: After an email and approval from the PI
